# Multi-Faceted Influence of Obesity on Type 1 Diabetes in Children – From Disease Pathogenesis to Complications

**DOI:** 10.3389/fendo.2022.890833

**Published:** 2022-06-16

**Authors:** Sebastian Ciężki, Emilia Kurpiewska, Artur Bossowski, Barbara Głowińska-Olszewska

**Affiliations:** Department of Paediatrics, Endocrinology, and Diabetology with Cardiology Division, Medical University of Bialystok, Białystok, Poland

**Keywords:** diabetes type 1, obesity, children, pathogenesis, complications, treatment

## Abstract

The prevalence of overweight and obesity among youth patients with diabetes type 1 is increasing. It is estimated, that even up to 35% of young patients with this type of diabetes, considered so far to be characteristic for slim figure, are overweight or even obese. General increase of obesity in children’s population complicates differential diagnosis of the type of diabetes in youths. Coexistence of obesity has clinical implications for all stages of diabetes course. It is confirmed that obesity is the risk factor for autoimmune diabetes, and is connected with the earlier onset of diabetes in predisposed patients. Many diabetic patients with obesity present additional risk factors for macroangiopathy, and are recognised to present metabolic syndrome, insulin resistance, and typical for diabetes type 2 - polycystic ovary syndrome, or non-alcoholic fatty liver disease. The prevalence of obesity rises dramatically in adolescence of diabetic child, more often in girls. It has negative impact on metabolic control, glycaemic variability and insulin demand. The risk for microangiopathic complications increases as well. The treatment is difficult and includes not only insulinotherapy and non-pharmacological trials. Recently treatment of insulin resistance with biguanids, and treatment with typical for type 2 new diabetes drugs like GLP-1 analogues, SGLT-2 receptor inhibitors, or even cases of bariatric surgery also has been reported.

## Introduction

Over the past few decades obesity became a worldwide epidemic (1) not only in adults but also in paediatric patients (2). According to WHO, in 2016 over 340 million of youth aged 5-19 were overweight or obese as were 39 million children under the age of 5 in 2020. This pandemic has not spared individuals with type 1 diabetes mellitus. Thus far, patients suffering from T1D usually were considered as lean, whereas those with type 2 diabetes generally exhibit overweight and obesity, yet growing number of recent studies have shown that the prevalence of the problem of excessive body weight is increasing in individuals with T1D (3–8). This type of disease is caused by progressive, irreversible autoimmune destruction of β-cells, leading to a total insulin deficiency and additionally, is more and more prevalent in the younger age groups. It was estimated that around 108.300 youths under 15 years were diagnosed with T1D in 2021 (9). It follows that both excessive body mass and T1D are increasing problems in population. Furthermore, their overlapping may cause significant health consequences.

Excessive body weight has been linked to T1D from its very beginning, numerous studies presumed its role in autoimmune diabetes pathogenesis (10, 11). Apart from a great number of environmental factors of overweight and obesity in T1D such as dietary mistakes, fear of insulin induced hypoglycaemia resulting in excessive carbohydrates intake and lack of exercise, sedentary lifestyle (12), authors frequently list modern patterns of intensive insulin therapy itself (13–15). Obesity can contribute to the challenges in attaining optimal glycaemic control (16). Excessive body weight in T1D patients predispose to the increased risk of the development of some serious health conditions like metabolic syndrome, cardiovascular or kidney diseases, thus increasing morbidity and mortality, causing the reduced life expectancy. Moreover, increased BMI which increases insulin resistance in combination with T1D may lead to the development of so-called double diabetes (17). General increase of obesity in children’s population complicates differential diagnosis of the type of diabetes in youths. The problem of obesity existing among youths with T1D looks definitely more like outbreak than occasional issue as up to 30-40% of young patients with T1D are recognised to be overweight or obese (4, 8, 18).

The aim of the present review is to summarize the most actual knowledge of the aetiology and repercussions of obesity in paediatric patients with T1D from diabetes pathogenesis to its complications, along with treatment directions that can withhold or counteract them.

## Obesity as a Pathogenic Factor of Developing T1D

### Complex Aetiology of T1D

The pathogenesis of T1D is very complex and depends on numerous heterogeneous aspects (19). Commonly mentioned are genetic, environmental, immune and recently added are microbiome factors (**Table 1**). HLA (human leukocyte antigen) located on chromosome 6 within the major histocompatibility complex (MHC) region - especially the HLA-DR3/4-DQ8 alleles are likely to be the main genetic risk factors responsible for the development of T1D by causing β-cell hyper-expression of MHC I presenting β-cell neoepitopes to cytotoxic T cells, hence β-cells destruction (20). Genes implicated in islet inflammation and beta-cell apoptosis play an important role in the onset of T1D. Thus far, genome-wide association studies (GWAS) have identified around 50 susceptibility loci for T1D (21). A large number of these candidate genes for example, IFIH1, PTPN2, CTSH, CLEC16A and also GLIS3 are expressed inside pancreatic islets and β-cells and are seemed to be involved in modulating the β-cells response to the immune system and viral infection and also in insulitis and apoptosis (22).

**Table 1:** Factors contributing to the development of T1D.

Factors contributing to the development of T1D	
**Genetic factors**	**Alterations in gut microbiota**
HLA-DR3/4-DQ8 Ins-VNTR polymorphisms CTLA-4 polymorphisms	nutrition, agricultural production, product preparation, personal hygiene, antibiotic use, caesarean section
**Immunologic factors**	**Environmental factors**
disturbed T-cells maturation and differentiation autoreactive T lymphocytes deficit in the Treg subpopulation autoantibodies: anti-GAD65, IA-2, IAA, ZnT8	viral infections molecular mimicry unhealthy lifestyle habits economic indicators
**Obesity (accelerator hypothesis)**
chronic low-grade inflammation self-tolerance mechanisms deficiency glucotoxicity insulin resistance

Immunologic factors include several abnormalities in maturation and differentiation processes of T-cells, which can cause T-lymphocyte escape central or peripheral tolerance induction (23). Some recent studies have demonstrated that patients with T1D present quantitative and qualitative deficit in the subpopulation of T regulatory lymphocytes, which may explain the limitless immune response, which eventually leads to autoimmunization (24). Apoptosis is the most likely way of insulitis and β-cell loss in T1D. One of the theories says that the autoreactive T lymphocytes within the islet microenvironment induce an inflammatory reaction with high levels of the proinflammatory cytokines, which stimulate the caspase cascade. Other hypotheses imply that apoptosis is induced through the perforating system or Fas/Fas ligand interaction directly by contact of autoreactive T lymphocytes with β-cells (24, 25). Autoantibodies against glutamic acid decarboxylase 65 (GAD65), tyrosyl phosphatase (IA-2), insulin (IAA) may prelude the onset of clinical manifestation of T1D for years, furthermore multiple islet autoantibodies are associated with highest risk of type 1 diabetes (26).

Environmental factors involved in the development of T1D include viral infections (27). Some studies have accumulated a lot of evidence supporting that enteroviral persistence in various tissues and its highly cytolytic activity can play an important role in some chronic diseases (28), while other have shown that congenital rubella can increase incidence of T1D in children (29). A hypothesis of molecular mimicry suggests that the immune response is targeted against autoantigens that mirror the viruses’ antigens resulting in cellular destruction. It has been showed that molecular mimicry with human cytomegalovirus, Coxsackie virus or rotavirus could promote autoimmunity to some islet antigens leading to autoimmunity in T1D (30–32). On the other hand, some experimental studies have suggested that it can’t be excluded that enteroviruses would be protective against T1D in certain conditions (33). There are also some evidences that COVID-19 may be a trigger for the development of diabetes as well as a factor that worsens complications in patients with existing diabetes (34–36). Further studies should take identifying the viruses connected with the occurrence of T1DM and determining their participation to pathogenesis of the disease into careful account.

There were some perturbations that vaccinations might be associated with the following development of chronic autoimmune diseases, T1D inclusive (37, 38). However, studies in a cohort of 584,171 participants have exhibited that recommended immunization schedule had no direct correlation with the incidence of T1D in children (39). Moreover, vaccines are extensively recommended in youths with T1D because it is a disease with an elevated risk of infection due to poor glycaemic control (40). Nevertheless, future researches are required to thoroughly determine the problems correlated with vaccine administration in patients with T1D.

Another environmental risk factors of the development of T1DM are lifestyle habits. Although, sedentary lifestyle, physical inactivity, high in fats and carbohydrates diet or smoking are factors contributing mainly to the development of type 2 diabetes and in general, they are widely known for increasing BMI (41), there were some studies demonstrated that high energy intake, especially rich in protein, fat, nitrosamine and carbohydrates, particularly disaccharides and sucrose, as well as more rapid growth in childhood or a larger relative body size, in both length and fat mass, were independently associated with the ongoing β-cell destruction and lead to an earlier clinical presentation of type 1 diabetes (42). Moreover, over-nutrition in childhood, hence increased childhood body size may rise T1D risk (43).

Human gut microbiota is another possible factor worthy of attention. Microbes colonizing the human gut feature in metabolic ailments and by extension seem to play an important role in the development of both diabetes and obesity, with autoimmune background. Human microbiota impact many aspects such as gut permeability, inflammatory responses, nutrient absorption, lipid metabolism, polysaccharide breakdown and bile acid modification. Studies have implied that T1D children show the increased Firmicutes to Bacteroidetes ratio, positive correlation of Bacteroides abundance with the presence of autoantibodies and negative correlation of Faecalibacterium abundance with HbA1c (44). Moreover, in the children with diabetes the quantity of essential to maintain gut integrity bacteria was significantly lower compared to their healthy controls (45). Decreased microbial diversity is believed to be associated with the T1D development, reported both in T1D patients (46) and in autoantibody-positive children (47). Changes in nutrition, agricultural production, product preparation, personal hygiene and antibiotic use, especially during the first years of life can change the composition of the gut microbiota (48). Increased risk of T1D in children born by caesarean section was suggested in conjunction with lack of contact with the mother’s vaginal microbiome hence, following distinctions in their gut microbiota due to abnormal colonization (49, 50). Recent investigations have proposed that the intestinal microbiome plays a key role in the mechanisms behind a proinflammatory issue that results in the destruction of pancreatic islet β-cells and loss of insulin generation in T1D (51). Nevertheless more studies are required to examine the complex role of human gut microbiota in the pathogenesis of T1D and to develop strategies to control the development of the disease.

The most frequently mentioned risk factors of T1D were briefly discussed, yet many authors suggest that also obesity itself has an impact of the development of T1D (**Table 1**). Obesity results in deficiencies of the human self-tolerance mechanisms by triggering chronic low-grade inflammation, reducing regulatory B as well as T cells, further resulting in increased Th17 and Th1 cells, creating the sublime milieu for the development of autoimmune disorders (10). Corpulent children with new-onset T1D exhibited a paradigm of adipokines and cytokines that suggest a proinflammatory state, which may lead to T1D onset and its complication (52). Studies have shown that genetic susceptibility in combination with a high-fat diet leads to the development of a T1D-like phenotype that is characterized by mononuclear cell infiltration and insulitis, surprisingly only in male examinees (53). A meta-analysis of nine studies has provided comprehensive evidence of a connection between childhood obesity and subsequent risk of diabetes with an OR of 1.25 and 2.03 (54). Higher BMI is believed to be an environmental accelerator which may contribute to the noticeable increase in both type 1 and type 2 of diabetes in childhood and younger age of onset (55). However, within the SEARCH cohort this dependence was seen only in children with fasting C-peptide levels below the median (56). A prospective cross-sectional study in a large cohort of T1D youth has also demonstrated that central obesity is correlated with earlier onset of the disease (57).

Nonetheless, because the aetiology of T1D is complex, it can be expected that the data presented above might be discussed. On the contrary, a case-control study has showed that high birth weight but not alone excessive weight gain prior to manifestation is related to earlier onset of diabetes in childhood (58). In a study of 777 children from the BABYDIAB cohort, insulin sensitivity and BMI-SDS were similar in both autoantibody-positive and -negative children (59). In the TrialNet Pathway to Prevention cohort, despite more children with excessive weight were determined to be as a single autoantibody-positive than those in the normal range of weight, they have found no convincing evidence in relation between conversion from single autoantibody to multiple autoantibodies or progression to T1D and BMI (60). The incidence of T1D is parallel to the increase in overweight and obesity, particularly in children with lower risk of developing the disease, i.e. older age children without high-risk HLA haplotypes- HLA DR3-DQ2 or DR4-DQ8 (61). Although evidence of potential epigenetic modifications of gene expression such as DNA methylation, underlines the need to define the role of external factors and their influence on gene expression in autoimmune diseases (62, 63). Attention is drawn to the fact that genes do not evolve so quickly and no other known environmental factor has a similar to obesity increase in the incidence of T1D.

### The “Accelerator Hypothesis”

In 2001, Wilkin T.J. published a theory that the divergence between an insulin-dependent (type 1) and a non-insulin-dependent diabetes mellitus (type 2) blurs and considers overlay rather than overlap of these two types. Decreased glucose control and hence rising blood glucose is believed to be a result of weight gain induces an increase in insulin resistance. Glucotoxicity accelerates apoptosis of β-cells by which activates their immunogens, either promotes autoimmunization in genetically predisposed individuals (64). In fact, it differentiates both types of diabetes only by tempo of changes and perceives them as a continuum, where the fluctuations between genetic response and insulin resistance evaluate the age of critical β-cell destruction along with clinical presentation (65).

A large number of studies have been performed to investigate the accelerator hypothesis. The ‘obesogenic’ environment which promotes insulin resistance could explain rising prevalence of T1D in people with insulin deficiency and/or islet autoimmunity (66). In the retrospective analysis of a cohort of 9,248 German and Austrian children with type 1 diabetes mellitus (67) as well as in Kibirige M. Report (68), BMI was inversely associated with age at diabetes onset. Nonetheless, higher BMI at T1D onset and the observation that pre-diabetic children are heavier and more insulin resistant than their peers may imply convergence of type 1 and type 2 diabetes phenotypes (69). On the other hand, retrospective data from 2020 have demonstrated no evident tendency between increasing of type 1 incidence in the ethnically homogenous paediatric population of Lesser Poland and younger age of diagnosis along with higher BMI-SDS over the study period. Only 2.7% of children were obese, 5.7% exhibited underweight when 91.6% presented BMI-SDS within the normal range at the time of diagnosis (70). The control of weight gain, and by extension insulin resistance, could be the means of minimising both obesity and diabetes. Further studies to determine the role of capability for prevention of type 1 diabetes based on avoidance of extensive body mass are needed.

## Obvious and Less Obvious Causes of Obesity in Type 1 Diabetes

Excessive weight in type 1 diabetes results from various complex factors. Insulin replacement therapy is believed to contribute most to weight gain in people with type 1 diabetes (71), but it can also be affected by their behaviour, lifestyle choices, psychosocial factors and fear of hypoglycaemia (72). Independent factors such as age, sex and duration of disease also play a role in development of overweight/obesity (73).

Intensive insulin therapy used in children with type 1 diabetes allows to maintain near-normoglycaemia and thus avoid long-term complications of the disease, what is the primary purpose of treatment (74). In spite of this, it is a key factor that contributes to obesity in patients with T1D (75). Results of Diabetes Control and Complications Trial (DCCT) indicated that during the first year of intensive insulin therapy subjects gained more weight (5.1 ± 4.6 kg) than participants treated conventionally (2.4 ± 3.7 kg) (76), and over the course of 6.5 years of the same study about 25% of subjects who were treated with intensive insulin therapy gained more weight than group treated in conventional way what resulted in occurrence of obesity (77). After that, Epidemiology of Diabetes Interventions and Complications (EDIC), the follow-up to DCCT demonstrated that subjects continued to gain weight. Their waist circumference and insulin dose also increased in the course of the study (78). In contrast, the Kaminsky and Dewey study showed that in spite of the risk of weight gain due to intensified insulin therapy, the BMIs of adolescents with T1D and a control group without chronic disease were similar, but it is worth noting that physical activity levels were also similar in both groups (79).

It is not fully understood how insulin therapy affects the weight gain, but there are several hypotheses. Patients treated with intensive insulin regime have significantly reduced HbA1c comparing to patients treated conventionally. Their blood glucose level falls below the renal threshold and consequently, glucosuria is almost completely eliminated. Their consumed calories are conserved, leading to reduced energy expenditure and weight gain (71).

It is extremely important to emphasize that supply of exogenous insulin does not exactly imitate endogenous one. Physiologically, insulin passes through the portal vein to the liver where it inhibits gluconeogenesis and then about 40-50% of insulin enters the systemic circulation. It acts on adipose tissue and muscle where it enhances glucose uptake and inhibits lipolysis. When exogenous insulin is administered subcutaneously, it enters the systemic circulation first and has a greater effect on adipose tissue and muscle than the liver. A disproportion between peripheral and hepatic insulin is created, which may lead to excess fat accumulation and peripheral hyperinsulinemia (71, 80).

Furthermore, bypassing the liver by exogenous insulin supply also has its consequences in impaired hepatic glycogenolysis and gluconeogenesis regulation during fasting and postprandial inhibition of glucagon secretion. This results in an inappropriate increase in glucagon levels and a decrease in stored glycogen in the postprandial state (81).

The DCCT study also showed that intensive insulin therapy leads to increased risk of hypoglycaemic episodes. What is already known, fear of hypoglycaemia is one of the factors causing weight gain in patients with type 1 diabetes. In an attempt to defend against the occurrence of hypoglycaemia (which remains the most common acute complication in people with type 1 diabetes), patients snack when they work out and overeat when hypoglycaemia occurs (71, 76). Moreover, they may prefer to wait to exercise until their blood glucose levels are in the proper range. Consequently, fear of hypoglycaemia during physical effort may have a negative impact on its frequency and quality (82).

The authors of The SWEET Registry demonstrated that switching from the multiple daily insulin injection to the continuous subcutaneous insulin infusion is significantly associated with an improvement in glycemic control but also with an increase in BMI in more than 4,000 youths with T1D (15). Interestingly, there are findings suggesting that in cases of co-occurring obesity and type 1 diabetes, making some changes to current therapy and using new long acting analogues as basal insulins, such as detemir, degludec and glar-300 may slightly prevent the weight gain associated with intensive insulin therapy (83). However, the study by Baskaran et al. showed that despite an increase in the use of intensive insulin therapy among paediatric patients with T1D from 52 to 97% between 1999 and 2009, the incidence of obesity/overweight in this group remained similar during these years (18, 82). Therefore, there must be other factors that have contributed to weight gain in these patients. Analysing further the problem, a sedentary lifestyle with lack of exercise, unhealthy eating habits and lack of sleep also play a role in weight gain.

Obesogenic environment with easy availability of processed, high-energy foods, poor dietary habits and sedentary behaviour are the main factors causing obesity in the general population and they contribute significantly to weight gain among patients with type 1 diabetes (83). It is worth noting that patients with T1D treated with intensive insulin therapy focus more on the amount of carbohydrates in a meal rather than on balanced nutrition. Because no food is prohibited, this results in the development of poor eating habits. Excessive amounts of fat (especially high saturated) and calories in their diet leads to weight gain and impaired metabolic control. In addition, some studies demonstrated that other eating behaviours like skipping breakfast and dinner may also contribute to overweight in young patients with T1D (12). Myśliwiec et al. in their study, which evaluated the dietary habits of adolescent males with type 1 diabetes, showed that a significant number of the subjects made many dietary mistakes, their diet was not well balanced and contained an excess of simple carbohydrates. Interestingly, most of the adolescents with type 1 diabetes who participated in the study had normal body fat distribution (84).

Regular physical activity is essential for young patients with T1D, and it has been proven to result in improved BMI, triglyceride, and cholesterol levels in this group. Unfortunately, young patients with T1D have less physical activity than those without the disease, and the previously mentioned fear of hypoglycaemia may contribute to this (12). In addition, Jamiołkowska-Sztabkowska et al. showed that regular physical activity contributes to longer partial remission time in children with newly diagnosed type 1 diabetes, resulting in better metabolic control of the disease in the long term (85).

Moreover, adolescents with T1D declare extended time spent in front of a screen (82). It was demonstrated that children with T1D who watched television during meals consumed more fat than children who did not, and girls with T1D who spent more time watching TV had a higher likelihood of being overweight (12).

Some researchers suggest that sleep deficiency may be related with overweight and obesity in youth with T1D. They sleep shorter than their healthy peers and have more periods of sleep apnoea. Disturbances in sleep architecture or sleep restriction may decrease insulin sensitivity and worsen diabetes compensation, thus inadequate amount of sleep has been linked to weight gain (82, 86).

Psychosocial factors such as depression, poor self-esteem, higher levels of stress, low social support or having a negative body image are believed to be associated with the occurrence of overweight or obesity, but this has not been intensively studied in relation to T1D (12).

It is noteworthy that eating disorders are more frequent in adolescents with type 1 diabetes than in their healthy peers. Anorexia nervosa, bulimia nervosa, binge eating or other specified feeding and eating disorders may be associated with this disease (87). A recent Danish national survey of adolescents with T1D found that about one-third of the subjects presented with symptoms of overeating and binge eating. Binge eating symptoms have been shown to be associated with lower quality of life, emotional problems and higher HbA1c and BMI-SDS (88). Furthermore, the study by De Keukelaere et al. revealed that increased age, being a female, longer time since diagnosis and diagnosis after puberty are independent factors associated with weight gain in children with T1D (73). The most common reasons of obesity in T1D are summarised in **Table 2**.

**Table 2:** Causes of obesity in children with type 1 diabetes.

Intensive insulin therapy	Factors related to the disease	Psychosocial factors
exogenous insulin has a greater effect on adipose tissue excess fat accumulation peripheral hyperinsulinemia impaired hepatic glycogenolysis and gluconeogenesis risk of hypoglycaemia	ncreased age at onset longer time since diagnosis diagnosis after puberty focus more on the amount of carbohydrates in a meal rather than on balanced nutrition fear of hypoglycaemia	depression poor self-esteem higher levels of stress low social support negative body image
Eating disorders	Obesogenic environment	Other
anorexia nervosa bulimia nervosa binge eating other specified feeding and eating disorders	sedentary lifestyle lack of exercise unhealthy eating habits high-energy foods	extended screen time sleep disturbances female sex

## Obesity as a Problem in Differential Diagnosis of Diabetes Types

In the past, diabetes diagnosed in childhood was almost always considered to be type 1. Nowadays, autoimmune-mediated type 1 diabetes still accounts for the majority of diagnoses in children. However, making an accurate diagnosis of the type of diabetes has become difficult, complex and complicated process, which may be challenging. Thus, the diagnostic approach of clinicians to children diabetes requires changes because clinical manifestations of the different subtypes of diabetes can overlap. Children with type 1 diabetes were for a long time considered to be thin at diagnosis, mainly due to the weight loss that usually occurs earlier. However, recent studies indicate an increase in BMI of children with type 1 diabetes. It must be highlighted that increasing prevalence of obesity in children results not only in their growing incidence of type 2 diabetes but also, and more frequently, in the development of a combined type 1 and type 2 diabetes (73, 89). Consequently, the presence of obesity creates new problems in the differential diagnosis of diabetes types.

Severe diabetic symptoms and significantly elevated glucose levels usually occur in individuals with type 1 diabetes. In addition, 40-60% of them are diagnosed with life-threatening diabetic ketoacidosis. Islet cells autoantibodies and autoantibodies to glutamic acid decarboxylase (GAD), the tyrosine phosphatases islet antigen 2 (IA-2) and IA-2β, insulin and zinc transporter 8 are autoimmune markers of this disease. According to the latest American Diabetes Association (ADA) clinical recommendations, the presence of multiple islet antibodies is considered to be a risk factor for clinical diabetes and their detection is recommended for screening for presymptomatic type 1 diabetes (90).

Therefore, type 1 diabetes in children can be predicted by the development of multiple islet autoantibodies. An interesting study by Ziegler et al. found that most children, who were at risk for type 1 diabetes and were diagnosed as having autoantibodies eventually developed the disease over the next 15 years (26). These findings seem to be confirmed by Gorus et al., who demonstrated that individuals with multiple autoantibodies develop symptomatic diabetes within 20 years; and this process is accelerated when IA-2A or ZnT8A are present (regardless of age, HLA-DQ genotype, and number of autoantibodies) (91). Hence, it is clear that the presence of autoantibodies is inseparably associated with type 1 diabetes.

According to ADA, screening for type 2 diabetes should be considered in children and adolescents after the onset of puberty or after age 10 years (depending on which occurs first) who are overweight or obese with one or more risk factors for diabetes (maternal gestational diabetes, a family history of type 2 diabetes in a 1st or 2nd degree relative, a predisposing race, or signs or symptoms of insulin resistance). There are reports of the onset of type 2 diabetes before the age of 10, which can be linked to the presence of multiple risk factors. As recommended, a fasting plasma glucose, oral glucose tolerance test and HbA1c can be used to diagnose pre-diabetes or diabetes in children and adolescents (90).

It is important that MODY (maturity-onset diabetes of the young) should not be omitted in the differential diagnosis of diabetes in children. It belongs to the group of monogenic diabetes, resulting from mutations of a single gene that is involved in the functioning of the pancreatic β-cells. It is inherited in an autosomal dominant manner, responsible for 1-5% of all diabetes cases and is typically diagnosed between the second and fifth decades of life. However, the estimated incidence of MODY in youth under 15 years of age with new-onset diabetes is 2.4%. There are 14 subtypes of MODY depending on the gene affected (92). It is relatively difficult to distinguish MODY from type 1 and type 2 diabetes using clinical characteristics. In contrast to type 1 diabetes, patients with MODY have preserved pancreatic β-cells function and their diabetes is well controlled with no or low doses of insulin for at least 5 years after diagnosis. On the other hand, the clinical features of type 2 diabetes are similar to MODY, but in most cases people with type 2 diabetes are obese or overweight, while MODY diabetes is not associated with increased body weight. However, both type 2 diabetes and MODY patients have a family history of diabetes (93). Interestingly, the American Diabetes Association recommends that children and young adults without typical features of type 1 or type 2 diabetes and with a family history of diabetes in successive generations should undergo genetic testing for MODY (90).

As mentioned, there are many clinical and diagnostic features that can be used to determine the type of diabetes in youth, such as severity of symptoms, age at onset of the disease, family history of diabetes, occurrence of overweight/obesity, C-peptide levels, islet antibodies and markers of insulin resistance. Some mentioned markers are more useful than others, and they may be helpful in identifying the type of diabetes (94, 95). But as mentioned earlier, clinical features at onset can overlap - obesity and ketoacidosis can occur in both type 1 and type 2 diabetes as well as age at diagnosis does not accurately differentiate diabetes types (96).

Patients with type 1 diabetes are usually described as those with low or normal weight, with little tendency to develop metabolic syndrome or insulin resistance (97). The prevalence of obesity and the presence of markers of insulin resistance are typically associated with people with type 2 diabetes (94). Moreover, the presence of islet antibodies can be found in most patients with T1D, while in patients with T2D they are usually absent (94). The presence of positive islet antibodies is recognised as factor determining autoimmune origin of disease, yet there exists the subfamily of type 2 diabetes with positive islet antibodies, being neither typical type 2 nor type 1, or quite opposite being both type 1 and type 2 (98). It must be emphasised that the boundaries between these types are blurring.

The increasing prevalence of obesity and overweight among patients with type 1 diabetes (T1D) has resulted in the emergence of double diabetes - new term which characterizes individuals with T1D who show clinical signs of type 2 diabetes (T2D) - obesity and insulin resistance (99). We may suspect double diabetes in a paediatric patient who has features typical of both T1D and T2D - when antibodies to β-cells are found in a child with T2D, or when a child with T1D is overweight/obese (96).

Furthermore, ADA in their latest guidelines recommends that overweight or obese children and adolescents who are suspected of having type 2 diabetes should be tested with a pancreatic autoantibody panel test to ensure that they do not suffer from type 1 diabetes (100).

It is extremely important fact that C-peptide, produced in equal amounts with insulin and used in assessing endogenous insulin secretion plays a large role in determining the type of diabetes. The presence of sustained insulin secretion and thus, C-peptide 3-5 years after diagnosis may indicate type 2 diabetes. When levels of C-peptide are low due to insulin deficiency, it may suggest the occurrence of type 1 diabetes. Clinicians must be careful when interpreting higher results, especially in obese individuals or those with signs of insulin resistance, because obese, insulin-resistant patients may have normal or elevated C-peptide levels at disease recognition, even if they have autoimmune-related type 1 diabetes and they will develop complete insulin deficiency in the future (101).

Szypowska et al. indicated a positive correlation between fasting C-peptide levels and BMI-SDS. In this study, 11.3% of the children with newly diagnosed type 1 diabetes were overweight or obese, and the median fasting C-peptide level in this group was higher than in normal- or underweight subjects. Consequently, the C-peptide levels of obese or overweight children with type 1 diabetes at the initial stage were preserved to a large extent (102).

Interestingly, study by Buryk et al. confirmed the association of an autoimmune process with the occurrence of insulin-requiring diabetes in children, but independently of obesity or the presence of autoantibodies. The authors connected obesity and diabetes-related autoimmunity with an enhanced T-cell response particularly in those with the highest levels of body fat and/or insulin resistance. Importantly, there are patients with autoimmune-related diabetes, at a point in their disease, when only T cells and not yet antibodies are measurable. According to the study, when comparing insulin resistant with insulin sensitive subject at the same point in their course of autoimmune progression, such insulin levels that are inadequate to maintain euglycaemia will appear earlier in the insulin resistant individual. The study also revealed that individuals with positive T-cell reactivity, but without the presence of conventional autoantibodies, were the group with the highest BMIz. Consequently, group of patients who may be classified as having type 2 diabetes because of the lack of autoantibodies, actually have diabetes associated with autoimmunity by T cells (103).

In conclusion, the occurrence of obesity in children with diabetes favours the overlap of clinical manifestations of different types of this disease. This creates a significant problem for clinicians, as it is more difficult for them to make a proper diagnosis. Recent studies have shown that clinical and diagnostic features previously used to differentiate between types of diabetes, such as C-peptide levels, weight on admission, presence of markers of insulin resistance or even the presence of islet antibodies, may lose their value in the face of the increasing prevalence of obesity in children.

## Obesity as a Factor that Alters/Disrupts the Well-Known Course of Type 1 Diabetes After Diagnosis

Most patients with long-duration T1D continue to secrete low levels of endogenous insulin yet as mentioned before, overweight and obese children exhibit higher levels of C-peptide than their lean peers. Usually, this preserved β-cells function means that patient achieves a good glycaemic control with low insulin demand. Interestingly, it was observed that the degree of metabolic control in individuals with greater weight gain was similar to those who maintained a stable weight, but at the expense of a higher daily insulin dose (78). Across two large registries in the US and Europe, higher BMIz was demonstrated to be significantly related to greater HbA1c levels and more incidents of severe hypoglycaemia (16). This apparent paradox may be due to the fact that healthy adipose tissue is essential for β-cell function. Likewise, while residual C-peptide secretion was proved to be correlated with lower risk of severe hypoglycaemia in T1D,people with higher BMI present this complication more often, presumably due to the fact that excessive weight impacts on impaired awareness of hypoglycaemia. The causes of this phenomenon are not completely understood, so it requires further investigation (104).

Additionally, Lee and co‐authors described that patients with higher BMI were characterized by increasing HbA1c together with weight gain (105). A prognostic model that included BMI, immunological markers, and age has predicted exacerbation of T1D defined by diminished residual fasting C-peptide after 1 year of follow-up [AUC of 0.936] (106). It suggests that obesity may be also qualified as a T1D progression predictor.

## Obesity as a Factor of Developing T1D Vascular Complications

Although type 1 diabetes is a treatable disease and despite advances in its treatment, it still remains a huge burden for patients. It carries the risk of serious complications, beginning with the occurrence of episodes of hypoglycemia or ketoacidosis and ending with long-term micro- and macrovascular complications. Microvascular complications are usually manifested in retino-, neuro- and nephropathy, but can also have an impact on cognitive function, the heart and other organs. In turn, conditions such as atherosclerosis, thrombosis in the heart, peripheral arteries and brain are considered to be macrovascular complications of type 1 diabetes. It is extremely important that cardiovascular disease remains a main cause of premature morbidity and mortality, resulting in an 8-13-year shorter life expectancy for people with type 1 diabetes compared to people without this condition (107). Atherosclerosis at the endothelial level begins early in patients with T1D even though coronary, cerebrovascular and peripheral artery disease does not manifest until adulthood. Therefore, patients with T1D diagnosed in childhood life present a high risk of premature development of cardiovascular disease thus maintaining good metabolic control throughout childhood and adolescence is essential for their future health and quality of life (108, 109). According to Merger et. al, the occurrence of metabolic syndrome in type 1 diabetes is associated with an increased incidence of micro- and macrovascular complications regardless of glycaemic control (110). Furthermore, youth with T1D and overweight or obesity have a higher likelihood of coexisting hypertension, abnormal lipids and elevated alanine aminotransferase compared to their healthy peers. It was also demonstrated that increased insulin resistance, which affects not only people with increased body weight without diabetes, but also individuals with type 1 diabetes and overweight or obesity, has a negative impact on the development of microvascular complications of type 1 diabetes and may be related to the occurrence of these macrovascular (71, 111).

SEARCH for Diabetes in Youth Study found that obesity, increased LDL cholesterol and triglycerides as well as lower HDL cholesterol are risk factors for diabetic peripheral neuropathy among youth with type 1 diabetes (112). This seems to be confirmed by a recent study by Franceschi et al., which showed that waist/height ratio and lipid disorders are one of the key risk factors not only for diabetic peripheral neuropathy but also for cardiac autonomic neuropathy for young people with type 1 diabetes (113). Furthermore, Price et al. in their study indicated obesity as the predominant risk factor for retinopathy and for cardiovascular disease (114). Some studies also demonstrated a link between kidney disease and obesity in both type 1 and type 2 diabetes and it was showed that high BMI ≥30 kg/m2 along with lipid disorders and elevated blood pressure are associated with an increased risk of albuminuria in adolescents and young adults with type 1 diabetes (115, 116). Interestingly, a group of children with diabetic nephropathy and coexisting type 1 diabetes participating in the Burlaka and Maidannyk study showed higher blood cholesterol levels and higher systolic and diastolic blood pressure values than children with type 1 diabetes without signs of nephropathy. Consequently, lipid disorders may be one of the factors that affect the kidney vasculature and blood pressure (117). In contrast, other studies have observed a reduced prevalence of micro/macroalbuminuria in obese children with T1D compared to those with healthy weight. Therefore, it cannot be ruled out that obesity carries some protective effect from micro/macroalbuminuria and this association requires further investigation (118).

It should be noted here that the American Heart Association identified type 1 diabetes as a high cardiovascular risk factor for paediatric patients, whilst obesity was placed in the “at risk” category. When obesity is severe, then it becomes a moderate risk factor for cardiovascular disease for these patients (119). Redondo et al. showed that there is an increased prevalence of cardiovascular risk factors in children with coexisting type 1 diabetes and overweight or obesity. In their study, obese children were more likely to be diagnosed with hypertension and dyslipidemia compared to those with healthy weight (3.5 and 2.2 times, respectively, after adjusting for age, sex, race/ethnicity, HbA1c and diabetes duration). Additionally, children with T1D and overweight had 1.4 times higher likelihood of having dyslipidemia despite rates of hypertension were similar to their normal weight peers (118). This seems to be confirmed by the recent study of Gomes et al. in which Brazilian adolescents with coexisting T1D and overweight/obesity had a higher prevalence of conventional risk factors for micro- and macrovascular complications of diabetes, such as duration of diabetes, hypertension, high LDL-cholesterol and presence of metabolic syndrome (120).

Although intensive insulin therapy has been proven to reduce the incidence of micro- and macrovascular complications of type 1 diabetes, its use is unfortunately associated with weight gain as a side effect of therapy. Therefore, its positive effects may be partially offset by the presence of obesity-related risk factors for cardiovascular disease. According to Purnell et. al, increased weight gain due to intensive insulin therapy is associated with central obesity, insulin resistance, progressive rise in blood pressure, dyslipidemia and increased measures of intima-media thickness and coronary artery calcium - subclinical markers linked to increased risk of cardiovascular disease (78, 108).

Atherogenic lipid profile connected with excessive body weight and also with unsatisfactory diabetes control in T1D patients are commonly known risk factor of CVD. This lipid profile is characterized by elevated levels of triglycerides, normal or slightly increased levels of LDL cholesterol and reduced levels of HDL cholesterol. The serum presence of dysfunctional HDL and small, dense LDL is prolonged and their binding to the arterial wall increases causing atherosclerotic changes (121). The prospective SEARCH study exhibited that the frequency of dyslipidemia with inadequate glycaemic control (HbA1c ≥ 9%), longer T1D duration, obesity, and hypertension correlated with arterial stiffness index and augmentation index (122). Moreover, dyslipidemia was observed in obese or overweight adolescents with well-controlled diabetes more often than in the group with insufficient diabetes control but normal BMI ranges (123). It shows that maintaining proper body weight in T1D patients is very important to reduce the risk of CVD.

## Obesity in T1D as a Cause of Additional Diseases

A large number of studies has demonstrated obesity as one of the prominent causes of negative health outcomes, which impacts physical wellness, by increasing the risk for the development of among others asthma, sleep apnoea, metabolic syndrome, osteoarticular and cardiovascular diseases, stroke or even certain types of cancers hence, higher total mortality, as well as sanity causing depression, decreased quality of life and poor self-esteem. Similarly to adults, obesity in children is correlated with analogous issues but in youth it may also cause femoral epiphyses or premature puberty. Additionally, poorer cognitive function, brain health, educational attainment may be more marked (124). All of listed above can be more dangerous for individuals with T1D because both obesity and T1D consequences can superimpose on each other leading to exacerbation of patients’ health condition (**Table 3**).

**Table 3:** Consequences of overlapping T1D and obesity complications.

T1D complications	Obesity complications	Consequences of overlapping
Macrovascular: atherosclerosis, thrombosis atherogenic lipid profile	Dyslipidemia Increased coronary artery calcium index Increased thickness of intima-media	Hypertension Increased risk of premature cardiovascular diseases Strokes
Hyperglycaemia	Insulin resistance Abdominal obesity Hyperlipidemia Hypertension	Metabolic syndrome
Hyperglycaemia	Adipose tissue dysfunction Elevated alanine aminotransferase	NAFLD-> CVD events, polyneuropathy, incidence of chronic kidney diseases
Impaired glucose tolerance Hyperinsulinemia	Dyslipidemia Insulin resistance Psychological comorbidities	PCOS: problems with fertility or higher risk for the development of endometrial cancer
Increased risk of nephropathy and albuminuria	High blood cholesterol Albuminuria	Kidney diseases, including end-stage renal disease

Another serious health issue correlated with both obesity and T1D is metabolic syndrome (MS). It has multiplicitous definitions, although is widely considered as a constellation of hyperglycaemia, high insulin resistance, abdominal obesity, hyperlipidemia, and hypertension (125). Metabolic syndrome or several of its components can be associated with chronic complications from diabetes, for example an increased chance for cryptogenic sensory peripheral neuropathy, which impacts small unmyelinated axons early in its course or even autonomic and large neurons later on (126). Even though metabolic syndrome tends to be manifested more frequently in people with diabetes than in general population, a significantly higher prevalence of its criteria was demonstrated in a group of overweight and obese than in normal weight T1D patients (127).

Non-alcoholic fatty liver disease (NAFLD), considered lastly as one of the components of MS, is a common chronic liver disorder that coexists in people with obesity and diabetes. Its prevalence increases with age, progress of metabolic diseases and BMI (128). Forasmuch as adipose tissue dysfunction that occurs in obesity is believed to be a key contributor to the pathogenesis of NAFLD, it is reasonable to anticipate a negative impact on liver health in obese T1D patients. Unfortunately, there have been no published studies that have compared the NAFLD phenotype between T1D individuals with normal and higher BMI ranges. NAFLD was demonstrated to be associated with an increased risk of CVD events (129), distal symmetric polyneuropathy (130) and incidence of chronic kidney diseases (131) in type 1 diabetic adults. New terminology updated from NAFLD- metabolic dysfunction-associated fatty liver disease (MAFLD) was established recently. The global prevalence of MAFLD estimated by repurposing existing data on fatty liver disease was 33.78% in the general population and 44.94% in obesity clinics (132).

The metabolic comorbidities such as insulin resistance, hyperinsulinemia, dyslipidemia or impaired glucose tolerance as well as the psychological comorbidities including anxiety, depression, eating disorders, low self-esteem, psychosexual dysfunction or poor quality of life occurring in both obesity and diabetes can lead to development of polycystic ovary syndrome (PCOS), characterized by ovarian dysfunction and hormonal imbalance, often starts during adolescence (133). PCOS appears frequently in adolescent girls with T1D because of exposing the ovaries and the adrenals to excessive insulin concentrations (134). It is an important consequence since women with PCOS exhibit problems with fertility or even higher risk for the development of endometrial cancer (135).

Additionally, a study in a large cohort of T1D patients has provided genetic evidence for a causal association between obesity and kidney diseases in T1D. Mendelian randomization analysis with a genetic risk score comprised of 32 validated BMI loci showed a U-shaped relationship between BMI over lifespan conferring an increased risk for the development of microalbuminuria, diabetic kidney disease and end-stage renal disease (136).

## Treatment of Obesity in T1D, a Pediatric Perspective

Because type 2 diabetes is more common than type 1, and the prevalence of overweight or obesity is higher in patients with T2D than in those with T1D, most weight control strategies have been described for people living with type 2 diabetes. However, it is not known if the same interventions are safe and effective for patients with T1D. These individuals face many difficulties when attempting to control their weight, such as experiencing hypoglycaemic episodes during fasting, dietary carbohydrate restriction or exercise (71) For instance, in order to maintain normal blood glucose levels, people with T1D often need a supply of simple carbohydrates, which do not have ideal nutritional value but instead are a source of extra calories. Therefore, the management of type 1 diabetes may be challenging and not compatible with the principles of healthy eating and active lifestyle that are the basis of optimal weight management (137). Many both pharmacological and non-pharmacological approaches to the treatment of obesity in type 1 diabetes are described below.

### Weight Management

In its latest guidelines, the American Diabetes Association (ADA) recommends weight control and weight loss in people with both types of diabetes or pre-diabetes and coexisting overweight or obesity (138). Many dietary interventions used for weight reduction have been studied in people with or without diabetes, but very few studies have focused strictly on patients with type 1 diabetes (99). It is known that reduction in total calorie intake plays a key role in weight loss in obese individuals, regardless of the occurrence of diabetes (83). Nevertheless, the recommended management of weight reduction in T1D is a low-calorie diet, which is based on the Mediterranean diet pattern, where 40-50% of energy comes from carbohydrates with a low glycaemic index and high content of fiber, 15-25% of energy comes from protein and 30-35% from fat (with high content of monounsaturated fats and low content of trans and saturated fats) (83). At this point it is worth mentioning that recently trending low carbohydrate or ketogenic diets should be used with caution in people with type 1 diabetes. Despite their potential to improve metabolic control, they may cause potentially dangerous side effects such as increased risk of diabetic ketoacidosis and deterioration of the lipid profile (139). Children and adolescents with type 1 diabetes should not significantly restrict carbohydrate intake, as this may adversely affect their growth, worsen the metabolic profile, increase the risk of developing eating disorders and hypoglycemia and impair the effect of glucagon in its treatment (140). Therefore, the ADA recommends an individualized approach to nutrition therapy for children and adolescents with T1D under the guidance of an experienced dietitian as an important part of their treatment plan (100).

### Physical Activity

Physical activity carries a number of physical and mental health benefits for youth with type 1 diabetes. Studies have shown that a combination of aerobics and strength training is beneficial in reducing not only BMI, but also HbA1c, triglycerides and total cholesterol in children with T1D. In addition, exercise significantly reduces depression, anxiety, and emotional disturbance, which may be important for youth with T1D who are at increased risk for depression (141). It has also been demonstrated that exercise increases lean body mass and energy expenditure at rest, reduces total and visceral adiposity and increases insulin sensitivity in children and adolescents (142). According to the recent American Diabetes Association (ADA) guidelines, all youth with type 1 diabetes are recommended to do 60 minutes of daily aerobic exercise of moderate- to vigorous-intensity and to perform vigorous muscle- and bone-strengthening exercises at least 3 times a week. To manage exercise-related hypo- and hyperglycemia, frequent measurement of blood glucose level before, during and after exercise is also recommended (100). Unfortunately, about two thirds of adolescents do not achieve the recommended time of physical activity. It is also a fact that children with type 1 diabetes are less physically active than their healthy peers (143). The main barrier for undertaking physical activity by these people appears to be the fear of hypoglycemia. Because of this, youth with T1D avoid or prematurely discontinue exercise or consume extra amounts of carbohydrates and calories, which eliminates the negative energy balance achieved through exercise (137). In parallel to carbohydrate consumption, insulin dose adjustment is a key factor in controlling blood glucose levels during and after exercise. Reducing insulin doses to protect against hypoglycemia induced by exercise is usually necessary for prolonged (more than 30 minutes) moderate-intensity activity. Fortunately, rapidly progressing diabetes technology helps patients with T1D to manage their blood glucose levels during exercise and gives them many tools, such as smartphone apps, insulin pumps, CGM and closed-loop technology (144). What is important, if proper measurements before exercise are carried out, patients with type 1 diabetes should be able to safely engage in both aerobic and weightlifting physical activities (99). Physical activity, which is an effective weight management intervention, presents many challenges for youth with type 1 diabetes and overweight or obesity. Thus, there is a need to develop safe strategies for undertaking physical activity in these patients.

### Multifactorial Approach to Obesity Prevention in Type 1 Diabetes

As mentioned earlier, the co-occurrence of obesity and type 1 diabetes in children increases the risk of developing vascular complications of diabetes and may also cause additional diseases. Therefore, early interventions are needed to maintain a healthy body weight in such patients. Prevention and management of excessive weight gain are crucial in the care of youth with type 1 diabetes. Guidelines concerning correct nutritional habits and physical activity for the entire family are required, because parents and family members have a significant impact on a child’s lifestyle. Thus, the family approach in the management of excessive body weight seems to be the most efficient, considering that parents are most often children’s role models who can influence their dietary choices and encourage them to exercise. In order to promote patient’s physical activity, advice needs to be given about their likely blood glucose responses. The counseling should include safety information and be suited to each person (140, 145). Hence, education of patients and their families on nutritional therapy and management of exercise-induced hypo- and hyperglycemia by qualified professionals is essential for self-management of the disease (146).

### Pharmacotherapy

Interestingly, it has been shown that insulin adjunctive therapies may have positive effects on weight management in people with type 1 diabetes, even though they were designed to improve glycaemic control (71). Although they are gaining popularity among adults, they have not been sufficiently researched with regard to children (137). The increasing prevalence of obesity additionally exacerbates the already existing insulin resistance among patients with T1D, which creates a need for adjunctive therapies in type 1 diabetes to reduce the risk of cardiovascular disease (147).

### Metformin

Metformin is a widely used oral antidiabetic drug that improves glycaemic in patients with type 2 diabetes in several ways, such as reducing glucose production in the liver and promoting glucose uptake in peripheral tissues, especially in the muscle (148). In adult patients with type 2 diabetes, this drug has been demonstrated to reduce the risk of cardiovascular disease and improve their body composition (147). Results from multiple studies show the promising potential of metformin as an insulin adjunctive therapy for poorly controlled overweight youth with type 1 diabetes to reduce cardiovascular disease risk (149). The findings of a randomized, double-blinded and controlled trial performed by Bjornstad et al. indicate that 3-month metformin therapy, which was additional to insulin administered to adolescents with type 1 diabetes mellitus, improved their insulin sensitivity and vascular health and also reduced their weight, body mass index and adiposity (147). These results are confirmed by the study by Libman et al. which also shows that adding metformin to insulin does not improve glycaemic control in overweight adolescents with type 1 diabetes after 6 months of such therapy (148). Consistent with these results are those by Nadeau et al. which also show a beneficial effect of metformin on insulin sensitivity, BMI and body composition in young people with type 1 diabetes (150). Metformin is found to be safe for adolescent children, with only a risk of minor gastrointestinal side effects, while acute side effects were not present in the conducted studies (149).

### GLP-1 Receptor Agonists and DPP-4 Inhibitors

GLP-1 is a hormone secreted from intestinal enteroendocrine cells. In a glucose-dependent manner it increases the secretion of insulin and inhibits the release of glucagon. It also delays gastric emptying and reduces appetite, leading to weight loss. It is inactivated by the enzyme dipeptidyl peptidase-4 (DPP-4) (72). GLP-1 receptor agonists such as exenatide or liraglutide are now more commonly used in the treatment of type 2 diabetes. These are not yet authorized for the treatment of patients with type 1 diabetes, but there is growing interest in their safety and efficacy as adjunctive therapy to insulin in patients with T1D (137). The primary mechanism of action of these drugs is to increase postprandial insulin secretion, which is not possible in patients with type 1 diabetes, but these patients may be able to take advantages from other effects provided by the drug (97). It has been demonstrated that both exenatide and liraglutide reduce A1C, body weight, fasting and postprandial blood glucose levels and insulin doses in adults with type 1 diabetes. At the same time, the risk of hypoglycemia in these patients is not increased, but there is an enhanced prevalence of gastrointestinal side effects (151, 152).. The results of the Traina et al. study, which investigated the use of once-weekly treatment with exenatide as add-on therapy to insulin for adult patients with uncontrolled type 1 diabetes, are consistent with this observation (153). Furthermore, Ghanim et al. in their study revealed that the use of liraglutide in adults with coexisting overweight or obesity and type 1 diabetes significantly reduces body weight (primarily through fat mass loss), improves the glycemic profile and lowers systolic blood pressure. Therefore, GLP-1 receptor agonists may be considered as adjunctive therapy to insulin in adult patients with type 1 diabetes, whereas there are no data regarding the use of these drugs in children (151, 154).

The review by Wang et al. indicates that DPP-4 inhibitors do not significantly reduce HbA1c levels or insulin dose and do not affect body weight and BMI in patients with T1DM (155). However, as GLP-1 receptor agonists show potential for the treatment of overweight and obesity in type 1 diabetes, more emphasis should be placed on research not only in adults but also in children and adolescents with this disease. Unfortunately, there is lack of data regarding the use of these drugs in this group.

### Pramlintide

As a synthetic analogue of amylin, a hormone that is secreted together with insulin, pramlintide supplements the action of insulin by decreasing postprandial glucagon output, delaying gastric emptying and promoting satiety (156). The Food and Drug Administration (FDA) has officially approved pramlintide as the only drug that can be used as a additional therapy to insulin to treat patients with type 1 diabetes in United States, but it should be noted that it is not approved for use in Europe (157, 158). Studies show that pramlintide as an add-on therapy to insulin for patients with type 1 diabetes brings beneficial effects such as improving glycaemic control, reducing insulin dose and body weight, while causing transient hypoglycemia and gastrointestinal side effects such as nausea, vomiting and anorexia at the beginning of treatment (159). There are limited data regarding the use of this drug in children with type 1 diabetes, but few studies show the beneficial effect of using such therapy in the pediatric population (160, 161).

### SGLT-2 Inhibitors

The sodium-glucose transporter SGLT-2, which is present in the proximal tubule and overexpressed in diabetic patients, promotes glucose reabsorption in this part of the nephron. Therefore, drugs that inhibit this transporter affect this process (83). SGLT2 inhibitors are available for the treatment of type 2 diabetes and the results of many studies have proven that they are safe and effective in treating this disease (157). In some countries, a few SGLT-inhibitors can be used as add-on therapy for patients with type 1 diabetes. Dapagliflozin has been approved for the treatment of adults with this disease in the United Kingdom, while the European Commission approved both dapagliflozin and sotagliflozin in 2019 for adults with T1D with BMI ≥ 27 kg/m2. In Japan, ipragliflozin can be used to treat such patients with T1D from 2018, while the United States FDA has not authorized any SGLT-2 inhibitor for type 1 diabetes patients (162).

The results of the meta-analysis conducted by El Masri et al. clearly indicate that SGLT2 inhibitor therapy in comparison to placebo resulted in a reduction in HbA1c, body weight, and total daily insulin dose in patients with type 1 diabetes. Since the combination of these drugs with insulin results in a reduction in total daily insulin dose, it may have the positive effect of reducing the frequency of dose-related insulin side effects such as hypoglycemia and weight gain (163). Importantly, the use of SGLT-2 inhibitors may be associated with the occurrence of euglycaemia diabetic ketoacidosis in both T1D and T2D patients. It is worth noting that type 1 diabetic patients treated with insulin pumps appear to be the highest risk group due to not taking long-acting insulin and sometimes experiencing infusion difficulties (164). In addition, other studies have shown an increased incidence of side effects, such as urinary tract and genital infections, which were associated with the use of these drugs (165). Unfortunately, published studies regarding the use of SGLT-2 inhibitors as an add-on therapy to insulin for the treatment of type 1 diabetes have focused mainly on the adult population (137), although in one study by Biester et al. indicated a potential benefit of using dapagliflozin in children over 12 years of age in reducing the average dose of insulin (166).

All of this points to the fact that the use of non-insulin drugs in the treatment of children with coexisting obesity and type 1 diabetes may have great potential for improving their glycaemic profile and reducing body weight. However, most of the studies are focused on the use of these drugs in adults, so further research is needed to provide evidence of the safety and efficacy of using insulin adjunctive therapies in the paediatric population.

### Bariatric Surgery

Bariatric surgery may be an option for people with type 1 diabetes who find it impossible to overcome obesity with the aforementioned methods (71). It has many advantages for individuals with T1D, such as weight reduction, lower total daily insulin dose, and less presence of with obesity comorbidities. However, it may have minimal impact on glycaemic control of these patients in the long term (167). Landau et al. conducted a study to examine the short- and long-term implications of bariatric surgery in patients with coexisting obesity and type 1 diabetes. They found that patients who underwent bariatric surgery experienced significant weight loss and improvements in both blood pressure and lipid profile. In contrast, the surgery did not result in improved glycaemic control in these patients. It should be noted that after surgery, 15% of subjects experienced diabetic ketoacidosis while 23% developed acute hypoglycemic episodes (168). Multiple studies confirm the occurrence of these side effects of bariatric surgery in patients with T1D (169). There are limited data on the outcomes of these surgeries in youth with type 1 diabetes. Nevertheless, there are two case reports describing the results of bariatric surgeries due to severe obesity in two adolescents with type 1 diabetes. The first obese male underwent a vertical sleeve gastrectomy. One year after surgery, he weighed 91 kg compared with 125 kg on admission. His total insulin requirement was lower as well, and one year after surgery there was a reduction in LDL levels from 180 mg/dl to 81 mg/dl and an increase in HDL levels from 32 mg/dl to 45 mg/dl. HbA1c levels remained unchanged. The second female patient with a primary diagnosis of type 2 diabetes treated only with metformin underwent Roux-en-Y gastric bypass surgery, and one month after surgery she developed diabetic ketoacidosis with the detection of positive islet antibodies, which turned out to be present also before surgery. As a result, the diagnosis was changed to T1D and the administration of insulin was started. 28 months after surgery, her BMI decreased by 42% and similar to the first patient, her lipid profile improved and her insulin requirement decreased. In contrast, her HbA1c level increased from 6.3% to 10% (170). The use of this method for the management of obesity in patients with type 1 diabetes requires further investigation to determine if the benefits of bariatric surgery outweigh its risks, such as occurrence of diabetic ketoacidosis and hypoglycemia, also in relation to paediatric population. A review of potential adjuvant pharmacological therapies to insulin in obese patients with type 1 diabetes is presented in **Table 4**.

**Table 4:** A review of potential adjuvant pharmacological therapies to insulin in obese patients with type 1 diabetes.

Name of the drug	Mechanism of action	The benefits for obese patients with T1DM	Side effects
**Metformin**	• Reducing gluconeogenesis in the liver, promoting glucose uptake in peripheral tissues	• Reducing cardiovascular risk, improving insulin sensitivity, reducing weight, BMI and adiposity	• Increased prevalence of minor gastrointestinal side effects
**GLP-1 receptor agonists**	• Increasing the secretion of insulin and decreasing the release of glucagon in a glucose-dependent manner	• Reducing HbA1c, BMI, fasting and postprandial blood glucose levels and insulin dose	• Increased prevalence of gastrointestinal side effects
**DPP-4 inhibitors**	• Inhibition of GLP-1 degradation	• No significant effect on the reduction of HbA1c, body weight, BMI or insulin dose	• Unknown
**Pramlintide**	• Decreasing postprandial glucagon output, delaying gastric emptying, promoting satiety	• Improving glycaemic control, reducing insulin dose and body weight	• Transient hypoglycemia, gastrointestinal side effects
**SGLT-2 inhibitors**	• Inhibiting glucose reabsorption in proximal tubule of nephron	• Reduction in HbA1c level, body weight and total daily insulin dose	• Euglycemic diabetic ketoacidosis, urinary tract and genital infections

## Conclusions

More and more children with T1D exhibit excessive body weight which previously was the domain of T2D patients. The prevalence of obesity in children with T1D creates many difficulties in the diagnostic process, mainly due to the overlapping phenotypes of both type 1 and type 2 diabetes, which has led to the distinction of the term double diabetes. It is worth adding that the coexistence of obesity and type 1 diabetes can alter or even disrupt the classic course of type 1 diabetes. Obesity can both contribute to the development of T1D and also be its result. Even though insulin is the treatment of choice in T1D, in addition to lack of physical activity, improper diet and fear of hypoglycaemia, it is also one of the factors contributing to weight gain in children with T1D.

Studies clearly show that, in comparison to their normal weight peers, overweight/obese children have a higher incidence of diabetes complications and further diseases resulting from them. Obesity in children with T1D should definitely be treated as early as possible to prevent especially cardiovascular risk factors, since cardiovascular diseases persist the leading causes of premature death among T1D patients. Although insulin therapy remains the most effective drug in improving glycaemic control in people with T1D, its use carries a risk of unexpected weight gain. Therefore, there is a need for new adjunctive drugs to insulin treatment that would help manage excessive body weight in patients with type 1 diabetes. Although such potential is demonstrated by drugs widely used in the treatment of T2D, there are still few reports describing the safety and efficacy of their use in people with T1D, especially in paediatric population.

It must not be forgotten that maintaining a healthy lifestyle at every stage of treatment remains a very important factor in the weight loss process. The problem of obesity in youth diagnosed with type 1 diabetes is a huge challenge for all patients, parents and diabetic medical teams, and everything points to the fact that this problem will continue to grow. Thus, most studies suggest that excessive weight gain reduces or nullifies the benefits of metabolic control. Consequently, further research is required to solve the issue of co-occurrence of obesity and type 1 diabetes in the paediatric population.

## Author Contributions

SC, EK - screened the literature search results and assessed for the eligibility for inclusion criteria, made substantial equal contribution to study design and conception, acquisition, analysis and interpretation of data, and wrote the original version of the paper. BG-O supervised the project, made substantial contribution to study design and conception, acquisition, analysis and interpretation of data, and revised the paper. AB was involved in the design, conception, analysis, and revised the paper. All authors contributed to discussion, read, and approved the final version of the manuscript.

## Conflict of Interest

The authors declare that the research was conducted in the absence of any commercial or financial relationships that could be construed as a potential conflict of interest.

## Publisher’s Note

All claims expressed in this article are solely those of the authors and do not necessarily represent those of their affiliated organizations, or those of the publisher, the editors and the reviewers. Any product that may be evaluated in this article, or claim that may be made by its manufacturer, is not guaranteed or endorsed by the publisher.
